# Generation of a highly attenuated strain of *Pseudomonas aeruginosa* for commercial production of alginate

**DOI:** 10.1111/1751-7915.13411

**Published:** 2019-04-21

**Authors:** Meagan E. Valentine, Brandon D. Kirby, Thomas R. Withers, Shannon L. Johnson, Timothy E. Long, Youai Hao, Joseph S. Lam, Richard M. Niles, Hongwei D. Yu

**Affiliations:** ^1^ Robert C. Byrd Biotechnology Science Center Progenesis Technologies, LLC One John Marshall Drive, Suite 314 Huntington WV 25755 USA; ^2^ Los Alamos National Laboratory Biosecurity and Public Health PO Box 1663 M888 Los Alamos NM 87545 NM USA; ^3^ Department of Pharmaceutical Science and Research School of Pharmacy Marshall University Huntington WV 25755 USA; ^4^ Department of Molecular and Cellular Biology University of Guelph Guelph ON Canada; ^5^ Department of Biomedical Sciences, Pediatrics Joan C. Edwards School of Medicine at Marshall University Huntington WV 25755‐9320 USA; ^6^ Present address: U. S. Food and Drug Administration Baltimore District/Morgantown Resident Post 604 Cheat Road, Suite 140 Morgantown WV 26508 USA; ^7^ Present address: Emmune Inc. 130 Scripps Way Jupiter FL USA

## Abstract

Alginate is an important polysaccharide that is commonly used as a gelling agent in foods, cosmetics and healthcare products. Currently, all alginate used commercially is extracted from brown seaweed. However, with environmental changes such as increasing ocean temperature and the increasing number of biotechnological uses of alginates with specific properties, there is an emerging need for more reliable and customizable sources of alginate. An alternative to seaweed for alginate production is *Pseudomonas aeruginosa*, a common Gram‐negative bacterium that can form alginate‐containing biofilms. However, *P. aeruginosa* is an opportunistic pathogen that can cause life‐threatening infections in immunocompromised patients. Therefore, we sought to engineer a non‐pathogenic *P. aeruginosa* strain that is safe for commercial production of alginate. Using a homologous recombination strategy, we sequentially deleted five key pathogenicity genes from the *P. aeruginosa* chromosome, resulting in the marker‐free strain PGN5. Intraperitoneal injection of mice with PGN5 resulted in 0% mortality, while injection with wild‐type *P. aeruginosa* resulted in 95% mortality, providing evidence that the systemic virulence of PGN5 is highly attenuated. Importantly, PGN5 produces large amounts of alginate in response to overexpression of MucE, an activator of alginate biosynthesis. The alginate produced by PGN5 is structurally identical to alginate produced by wild‐type *P. aeruginosa*, indicating that the alginate biosynthetic pathway remains functional in this modified strain. The genetic versatility of *P. aeruginosa* will allow us to further engineer PGN5 to produce alginates with specific chemical compositions and physical properties to meet different industrial and biomedical needs.

## Graphical abstract

This paper describes the development of a safe bacterial strain as a microbial cell factory for commercial production of alginate through a systemic approach to remove pathogenicity genes from the chromosome of *Pseudomonas aeruginosa*.

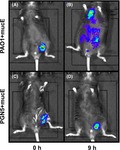

## Introduction

Alginate is a commercially important biopolymer produced by brown seaweed and some species of bacteria. The polymer is a polysaccharide composed of the uronic acid stereoisomers, α‐l‐guluronic acid (G) and β‐d‐mannuronic acid (M) (Haug *et al*., 1966). The relative abundance and distribution, as well as chemical modification of these monomers, determine the physical characteristics of the polymer. For example, regions enriched with repeating G residues (G‐blocks) result in a more rigid alginate structure, while alginate polymers with an abundance of M residues (M‐blocks) have a more flexible structure, and acetylation of M monomers increases fluid absorption by the polymer (Smithsrød and Whittington, 1969; Stokke *et al*., 1993; Vold *et al*., 2006). The industrial utility of alginate is primarily due to its biocompatibility and its ability to form sodium and calcium gels.

Alginate is a common additive to foods and cosmetics, often used as a gelling agent, and has many applications in the medical industry as well (Helgerud *et al*., 2009). For example, alginate hydrogels are used in advanced wound care dressings to absorb fluid, providing a moist environment to decrease infection risk and speed up wound closure (Paul and Sharma, 2004). Alginate is also used as an excipient for tablets and is a common bio‐ink used to form biocompatible, non‐immunogenic frameworks for the 3D printing of tissues and organs (Augst *et al*., 2006; Andersen *et al*., 2012; Lee and Mooney, 2012). New studies are highlighting exciting new health benefits of alginate, especially for its antioxidant and anti‐inflammatory properties (Liu *et al*., 2017; Qu *et al*., 2017; Mortazavi‐Jahromi *et al*., 2018; Taeb *et al*., 2018). For example, a recent study in osteoporosis patients with degenerative lumbar disease showed that oral administration of alginate nanoparticles after corrective surgery increased antioxidant and anti‐inflammatory agents such as superoxide dismutase (SOD) and interleukin‐1 receptor antagonist (IL‐1ra) while decreasing markers of inflammation and oxidative stress such as alanine aminotransferase (ALT) and interleukin 1β (IL‐1β) (Qu *et al*., 2017). Additionally, oral administration of alginate devoid of G residues (i.e. polymannuronic acid) to mice counteracted the inflammatory and obesogenic effects of a high‐fat, high‐sucrose diet, possibly through modulating the composition of the gut microbiota (Liu *et al*., 2017).

Currently, all alginate used commercially is extracted from brown seaweed (Peteiro, 2018). However, increases in ocean temperature and CO_2_ levels as a result of climate change are a looming threat to seaweed habitats and alginate yields. While laboratory cultivation of brown seaweed is possible, it is expensive. Additionally, the ability to tailor the composition of seaweed alginate (e.g. M:G ratio) to suit specific applications is limited. Thus, there is a growing need for more reliable and customizable sources of alginate.

The only other known sources of alginate are from cultures of bacterial species of the genera *Azotobacter* and *Pseudomonas* (Gorin and Spencer, 1966; Linker and Jones, 1966; Govan *et al*., 1981). Though the potential for commercialization of bacterial alginate has long been recognized and researched, seaweed remains the sole source of commercially available alginate (Rehm and Valla, 1997; Remminghorst and Rehm, 2006; Hay *et al*., 2013). Among the bacteria that produce alginate, alginate biosynthesis has been best studied in *Pseudomonas aeruginosa*. *P. aeruginosa* is a ubiquitous Gram‐negative bacterium found in soil, water and man‐made environments. However, it is also an opportunistic pathogen that can cause life‐threatening infections in immunocompromised patients, including those with respiratory infections, urinary tract infections, bacteraemia and underlying disease such as cystic fibrosis (Lyczak *et al*., 2002; Driscoll *et al*., 2007). *P. aeruginosa* has a large genome and harbours genes for the production of many secreted virulence factors, including proteases, exoenzymes, exotoxins and lipases (Gellatly and Hancock, 2013). In addition to secreted toxins, the cell envelope of *P. aeruginosa* contains virulence factors such as flagella, adhesins and lipopolysaccharide (LPS) in its outer membrane (Gellatly and Hancock, 2013). *Pseudomonas aeruginosa* is also capable of forming biofilms that protect the organism from environmental stressors, including dehydration, predation, antibiotics and host defences (Schwarzmann and Boring, 1971; Govan and Fyfe, 1978; Nivens *et al*., 2001; Pier *et al*., 2001; Leid *et al*., 2005). The *P. aeruginosa* biofilm is rich in alginate (Pedersen *et al*., 1990).

In *P. aeruginosa*, alginate is polymerized, modified and secreted by a multiprotein complex that spans the inner and outer membranes of the organism (Franklin *et al*., 2011; Hay *et al*., 2013). The proteins that make up this complex are expressed from the alginate biosynthetic operon, which contains 12 coding sequences (*algD*,* alg8*,* alg44*,* algK*,* algE*,* algG*,* algX*,* algL*,* algI*,* algJ*,* algF* and *algA*), as well as one separately localized gene (*algC*) (Chitnis and Ohman, 1993). These genes are highly conserved between bacterial species known to produce alginate. In *P. aeruginosa*, expression of this operon is controlled by the autoregulating sigma factor AlgU, which is expressed from the alginate regulatory operon (*algU*,* mucA*,* mucB*,* mucC*,* mucD*) (Deretic *et al*., 1994; Firoved and Deretic, 2003). AlgU is a major regulator of alginate biosynthesis, and its activity is highly regulated. A major regulator of AlgU is MucA, a protein that binds and sequesters AlgU to the inner membrane (Schurr *et al*., 1996; Xie *et al*., 1996; Mathee *et al*., 1997). Positive regulators of alginate biosynthesis usually act via degradation of MucA to release AlgU, leading to expression of the biosynthetic operon and alginate production.

Extracting and using alginate produced by *P. aeruginosa* is problematic due to the potential pathogenicity of the organism, as well as low and inconsistent alginate yields in wild‐type strains. Our group previously reported that overexpression of a novel activator of alginate biosynthesis, MucE, induces a mucoid phenotype and consistent overproduction of alginate by activating the protease AlgW to cleave MucA, thus activating AlgU (Qiu *et al*., 2007). We now report that by deleting five key virulence factor genes from the chromosome of a wild‐type *P. aeruginosa* strain, we have created a non‐pathogenic strain, PGN5. Significantly, this strain is still able to produce large quantities of alginate upon deliberate activation of the alginate biosynthetic pathway through MucE overexpression. We have collected structural data to show that alginate produced by PGN5 is identical to that of wild‐type *P. aeruginosa*. Therefore, PGN5 may provide a suitable alternative to seaweed for producing alginate that is safe for human use. Additionally, further genetic manipulation of PGN5 can potentially provide a reliable source of alginates and other recombinant proteins with specific physical properties to suit different industrial and biomedical needs.

## Results

### Deletion of five key pathogenicity genes in *P. aeruginosa*

**Figure 1:**
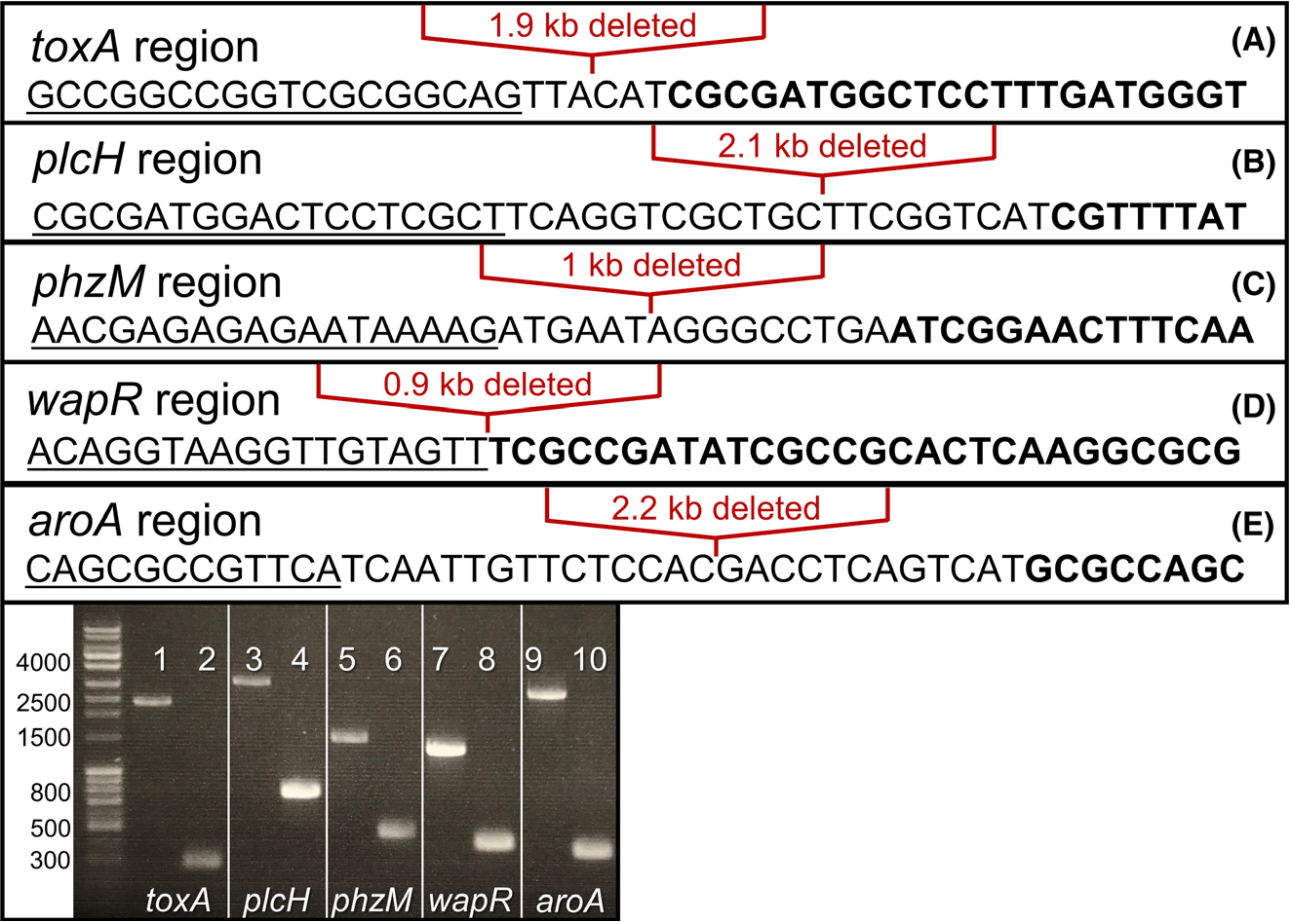
Confirmation of deletion of five gene sequences in *P. aeruginosa* strain PGN5. A–E. Sanger sequencing results of each gene deletion in the PGN5 strain. Panels A, B and E show plus strand sequence obtained. Panels C and D show minus strand sequence obtained. Underlined sequence is upstream of the start codon of each gene. Bold sequence is downstream of the stop codon of each gene. Location of deleted sequence is shown by red bracket. F. Image of agarose gel electrophoresis from PCR amplification of deleted gene regions. Odd lanes are PCR products from *P. aeruginosa* strain PAO1, and even lanes are PCR products from strain PGN5.

To generate an attenuated strain of *P. aeruginosa* for production of alginate, the following virulence factor genes were sequentially deleted from the chromosome of the wild‐type strain PAO1: *toxA*,* plcH*,* phzM*,* wapR* and *aroA*. *toxA* encodes the secreted toxin Exotoxin A, which inhibits protein synthesis in the host by deactivating elongation factor 2 (EF‐2) (Iglewski *et al*., 1977; Michalska and Wolf, 2015). *plcH* encodes the secreted toxin haemolytic phospholipase C, which acts as a surfactant and damages host cell membranes. *phzM* encodes phenazine‐specific methyltransferase, an enzyme required for the production of the redox active, pro‐inflammatory, blue–green secreted pigment, pyocyanin (Rada and Leto, 2013). *wapR* encodes a rhamnosyltransferase involved in synthesizing O‐antigen, a component of lipopolysaccharide (LPS) of the outer membrane of the organism. *aroA* encodes 3‐phosphoshikimate 1‐carboxyvinyltransferase, which is required intracellularly for aromatic amino acid synthesis. Deletion of *aroA* from the *P. aeruginosa* genome has previously been shown to attenuate the pathogenicity of this bacterium (Priebe *et al*., 2002). Each gene was successfully deleted using a homologous recombination strategy with the pEX100T‐Not1 plasmid (Schweizer and Hoang, 1995; Damron *et al*., 2009). The in‐frame, marker‐less deletion of these five gene sequences was verified by Sanger sequencing and by whole‐genome re‐sequencing (Fig. 1). We have designated this engineered strain as PGN5. The whole‐genome sequence of PGN5 has been deposited to NCBI GenBank with an accession number of CP032541. Overall, the PGN5 genome was similar to PAO1 (Fig. S1, Tables S1–S3). Gaps in the PGN5 sequence included regions of the genes *toxA*,* plcH*,* phzM*,* wapR* and *aroA* (Table S3).

**Figure 2:**
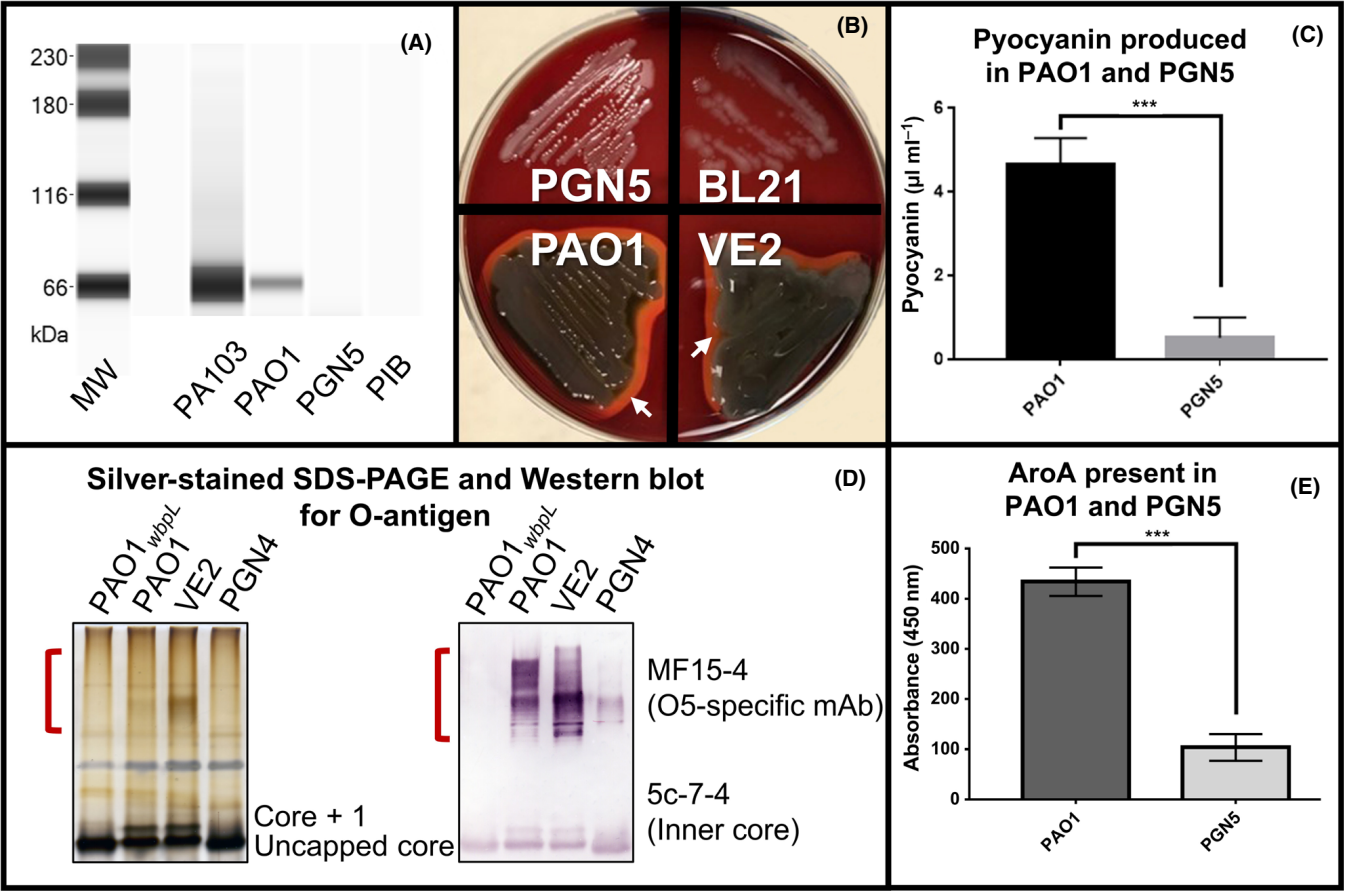
Confirmation of loss of deleted gene products in *P. aeruginosa* strain PGN5. A. Western blot analysis of Exotoxin A from the concentrated extracellular fraction of Exotoxin A‐positive *P. aeruginosa* PA103, wild‐type *P. aeruginosa* PAO1 and *P. aeruginosa* PGN5. PIB was used as a negative control. B. Haemolysis on blood agar observed with *P. aeruginosa* strains PAO1 and VE2, but not PGN5 or *E. coli* strain BL21. Areas of β‐haemolysis shown with white arrows. C. Pyocyanin concentrations detected in PAO1 and PGN5 media. D. Silver‐stained SDS‐PAGE (left) and Western blot analysis (right) against O‐antigen on cell lysates of *P. aeruginosa* strains PAO1_*wbpL*_, PAO1, VE2 and PGN4. O‐antigen regions indicated by red brackets. Antibodies used are indicated. E. ELISA of cell lysates of PAO1 and PGN5 for 3‐phosphoshikimate 1‐carboxyvinyltransferase (AroA). ***P < 0.001, determined by two‐tailed Student's *t*‐test.

**Figure 3:**
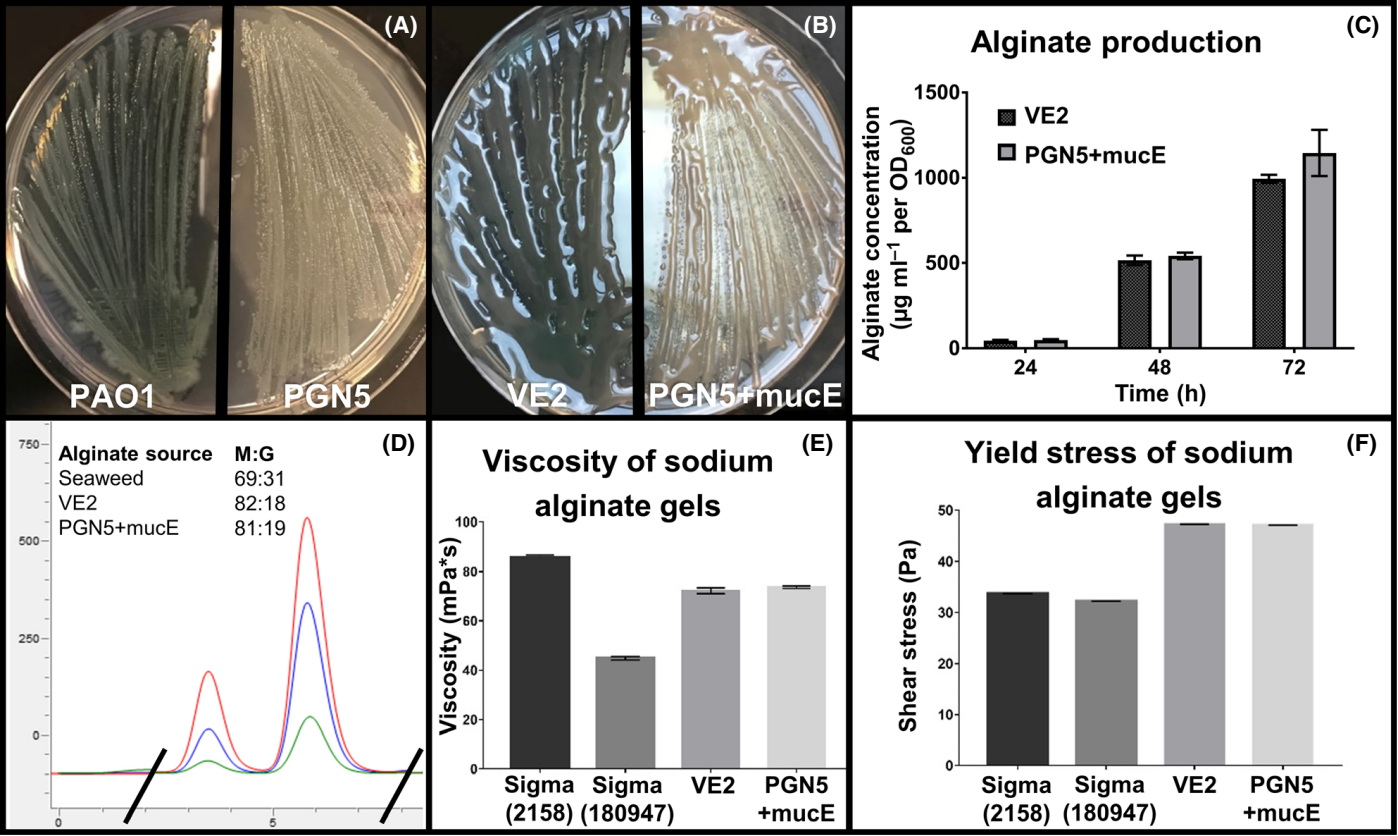
Phenotype and alginate characterization of *P. aeruginosa* strain PGN5. A. Non‐mucoid phenotype of *P. aeruginosa* strains PAO1 and PGN5 on PIA. B. Mucoid phenotype of PGN5+mucE and VE2 on PIAGm300. C. Uronic acid (carbazole) assay was used to measure alginate production on VE2 and PGN5+mucE over 72 h. Mean ± SD shown. D. Overlay of HPLC chromatograms of alginate prepared from a seaweed alginate control (red), and *P. aeruginosa* strains VE2 (blue) and>PGN5+mucE (green). Only area of interest is shown. Determined M:G ratios shown in table inlay. *X*‐axis = retention time; *Y*‐axis = absorbance units. E. Measured viscosity of 2% sodium alginate gels prepared from seaweed alginate samples Sigma 2158 and Sigma 180947, and alginate produced by VE2 and PGN5+mucE. Mean ± SD shown. F. Measured shear stress/shear rate measured from 2% sodium alginate gels prepared from seaweed alginate samples Sigma 2158 and Sigma 180947, and alginate produced by VE2 and PGN5+mucE. Mean ± SD shown.

To verify gene deletion and attenuation of the PGN5 strain, the presence of the products of the deleted genes was measured and was either undetectable, or significantly reduced in the PGN5 strain. To test for the *toxA* gene deletion in PGN5, we performed a Western blot analysis for the presence of Exotoxin A secreted into the culture medium. The extracellular fractions of PAO1 and PGN5 were similar in their banding profiles with a Coomassie total protein stain (Fig. S2). However, extracellular Exotoxin A was detected in wild‐type PAO1 control, but not in the PGN5 strain (Fig. 2A). To confirm the loss of *plcH*, we assessed haemolysis on blood agar. The haemolytic assay was carried out by streaking PAO1, PGN5, *P. aeruginosa* mucoid strain VE2 (i.e. PAO1+mucE) and a negative control, *Escherichia coli* strain BL21 on blood agar plates. A clear zone was observed surrounding PAO1 and VE2 cell growth, indicating complete (β‐) haemolysis (Fig. 2B). In contrast, the blood agar remained red and opaque surrounding PGN5 and BL21 growth, indicating negligible or no haemolytic activity in these strains (Fig. 2B). To assess for deletion of *phzM*, we extracted and measured the amount of pyocyanin secreted by PAO1 and PGN5. The amount of pyocyanin detected was significantly reduced in PGN5 (Fig. 2C). In fact, the difference in pigment production between PAO1 and PGN5 was immediately apparent on agar plates (Fig. 3A and B). To test for *wapR* gene deletion, we performed an LPS extraction, followed by silver‐stained SDS‐PAGE and Western blot on the following strains: PAO1, PGN4 (PGN5 without *aroA* deletion), VE2 and PAO1_*wbpL*_, which serves as a negative control due to a deletion in the O‐antigen ligase gene and thus produces no O‐antigen. The presence of O‐antigen was detected in PGN4, but the level of LPS banding was significantly reduced compared to the LPS banding profile observed in PAO1 and VE2 (Fig. 2D). Lastly, to test for *aroA* deletion, ELISA was performed to detect the presence of 3‐phosphoshikimate 1‐carboxyvinyltransferase in cell lysates prepared from PAO1 and PGN5. The ELISA results showed that the amount of 3‐phosphoshikimate 1‐carboxyvinyltransferase was significantly reduced in PGN5, compared to that in PAO1 (Fig. 2E). Additionally, the deletion of *aroA* resulted in slower growth in the PGN5 strain, a growth defect that was restored with the addition of 1 mg/mL of aromatic amino acids (W, Y, F) to the culture medium (Fig. S3), consistent with previous observations (Priebe *et al*., 2002).

### *P. aeruginosa* strain PGN5 produces a high amount of alginate

PAO1, the parent strain of PGN5, is a wild‐type *P. aeruginosa* strain that produces relatively small amounts of alginate and exhibits a non‐mucoid phenotype; thus, PGN5 is also non‐mucoid when cultured (Fig. 3A). In PAO1, the alginate biosynthetic operon, which contains genes required for alginate production, is negatively regulated. Activation of this operon leads to alginate production and a mucoid phenotype. For example, the *P. aeruginosa* strain VE2 (*i.e*. PAO1+mucE) displays high levels of alginate production and a mucoid phenotype (Fig. 3B) (Qiu *et al*., 2007). This strain was generated through a *mariner*‐based transposition mutagenesis on PAO1 and carries a gentamicin resistance cassette upstream of the *mucE* gene, which drives its overexpression. The mucoid phenotype in this strain is stable, with no observed reversion to non‐mucoidy after over 12 passages on non‐selective media. Thus, overexpression of MucE, an activator of the alginate biosynthetic pathway, induces a strong and stable mucoid phenotype in the PAO1 strain. The plasmid pUCP20‐pGm‐*mucE*, which constitutively overexpresses MucE, was used to test whether the genetically modified PGN5 strain could produce alginate (Qiu *et al*., 2007). Indeed, the presence of this plasmid in PGN5 (PGN5+mucE) induced a mucoid phenotype (Fig. 3B). Through over 12 passages on non‐selective media, the predominant phenotype of PGN5+mucE colonies is mucoid; however, some reversion to non‐mucoidy occurs. Thus, the mucoid phenotype in this strain is relatively less stable than VE2, presumably because overexpression of MucE occurs from a plasmid, rather than a chromosomal locus. To measure the amount of alginate produced by PGN5+mucE on a cellular level, we performed a standard carbazole assay, which showed that the PGN5+mucE and VE2 strains produce comparable amounts of alginate (Fig. 3C). Indeed, VE2 and PGN5+mucE produced averages of 24.49 ± 1.64 g l^−1^ and 26.41 ± 3.39 g l^−1^ of alginate (dry weight) respectively.

To examine whether the alginate produced by PGN5+mucE was similar in composition to alginate produced by VE2, we performed HPLC to compare the M and G content of alginate produced by each strain. The HPLC chromatograms obtained from alginate prepared from VE2 and PGN5+mucE were similar to each other and to a seaweed alginate control (FMC 8133) (Fig. 3D). The M:G ratios of VE2 and PGN5+mucE were consistently determined around 80:20, while seaweed alginate was consistently around 70:30. To confirm that the physical properties of VE2 and PGN5+mucE alginates were also similar, we prepared 2% sodium alginate gels from alginate produced by each strain, as well as two different commercial seaweed controls (Sigma 2158 and Sigma 180947) and measured the viscosity and yield stress. The average viscosities of VE2 and PGN5+mucE alginate gels were consistent and comparable at 73.58 and 72.12 mPa respectively (Fig. 3E). While the viscosities of seaweed alginate gels varied between samples, viscosities of VE2 and PGN5+mucE alginate gels were within the range of gels prepared from seaweed alginate (Fig. 3E). Likewise, the average yield stress of VE2 and PGN5+mucE alginate gels was similar at 47.34 and 47.16 Pa respectively (Fig. 3F). The average yield stress of sodium alginate gels prepared from Sigma 2158 and Sigma 180947 was 33.64 and 32.15 Pa respectively; thus, they have a lower tensile strength than gels prepared from VE2 and PGN5+mucE alginate (Fig. 3F).

### *P. aeruginosa* strain PGN5 did not cause mortality in mice

**Figure 4:**
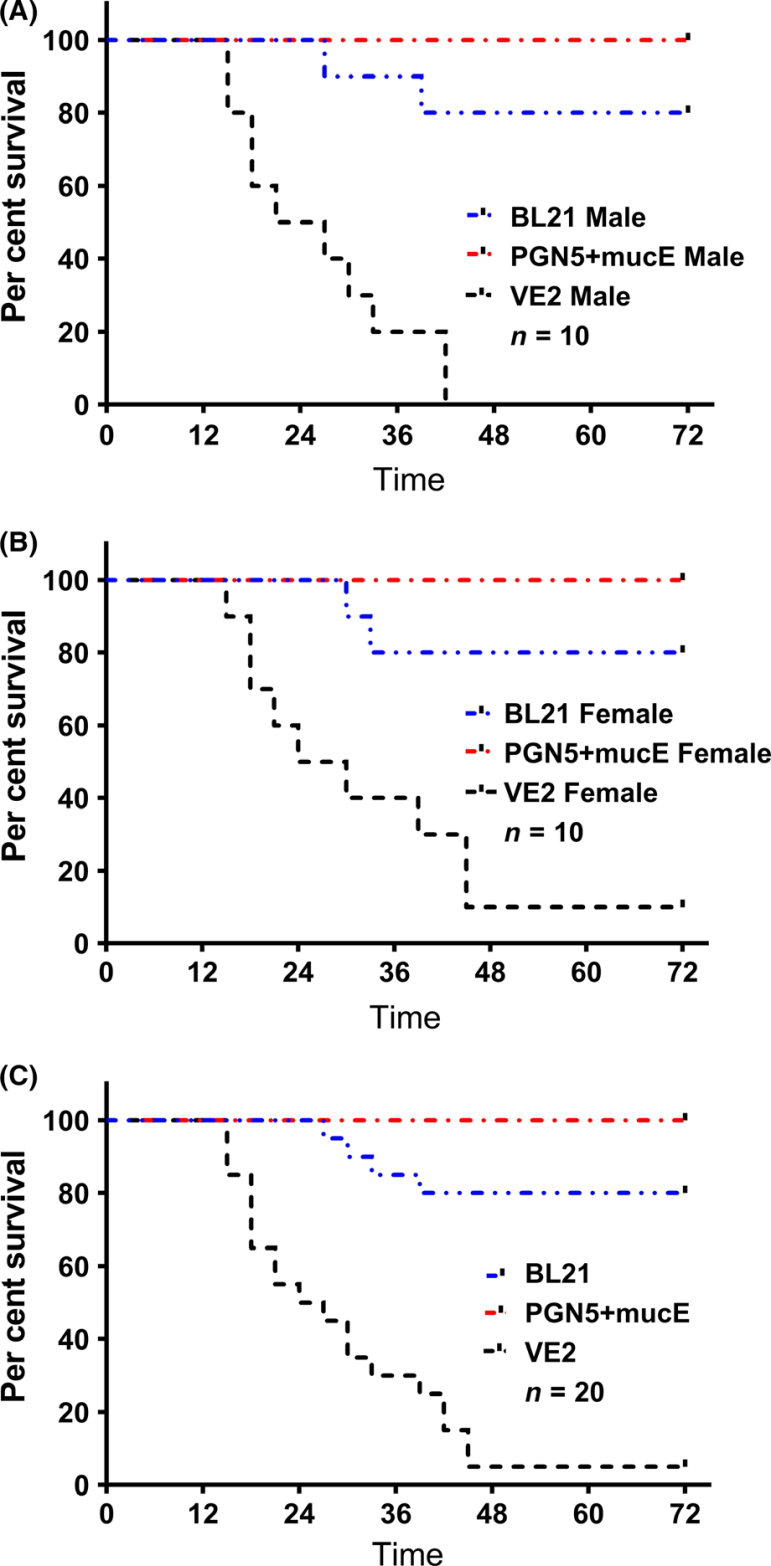
Per cent survival of male and female C57BL/6 mice after injections with *P. aeruginosa* strains VE2, PGN5+mucE or *E. coli* BL21. A. Per cent survival of male mice *n* = 10. B. Per cent survival of female mice, *n* = 10. C. Per cent survival of combined survival of both male and female, *n* = 20.

To test whether the pathogenesis of PGN5 was attenuated, C57BL/6 mice were challenged with intraperitoneal injection of 5 × 10^8^ cells of the PCR‐ and phenotype‐validated strains VE2, PGN5+mucE or *E. coli* BL21, or PBS as a negative control. Injection with the VE2 strain was fatal in 95% of mice within 48 h (Fig. 4). In contrast, injection with BL21 cells resulted in 20% mortality within 48 h, while no mortality was observed from injection with either the PGN5+mucE strain or PBS (Fig. 4). The mice were monitored for 4 weeks post‐injection, and no change in mortality was observed.

### A bioluminescent construct of PGN5 did not disseminate in mice

**Figure 5:**
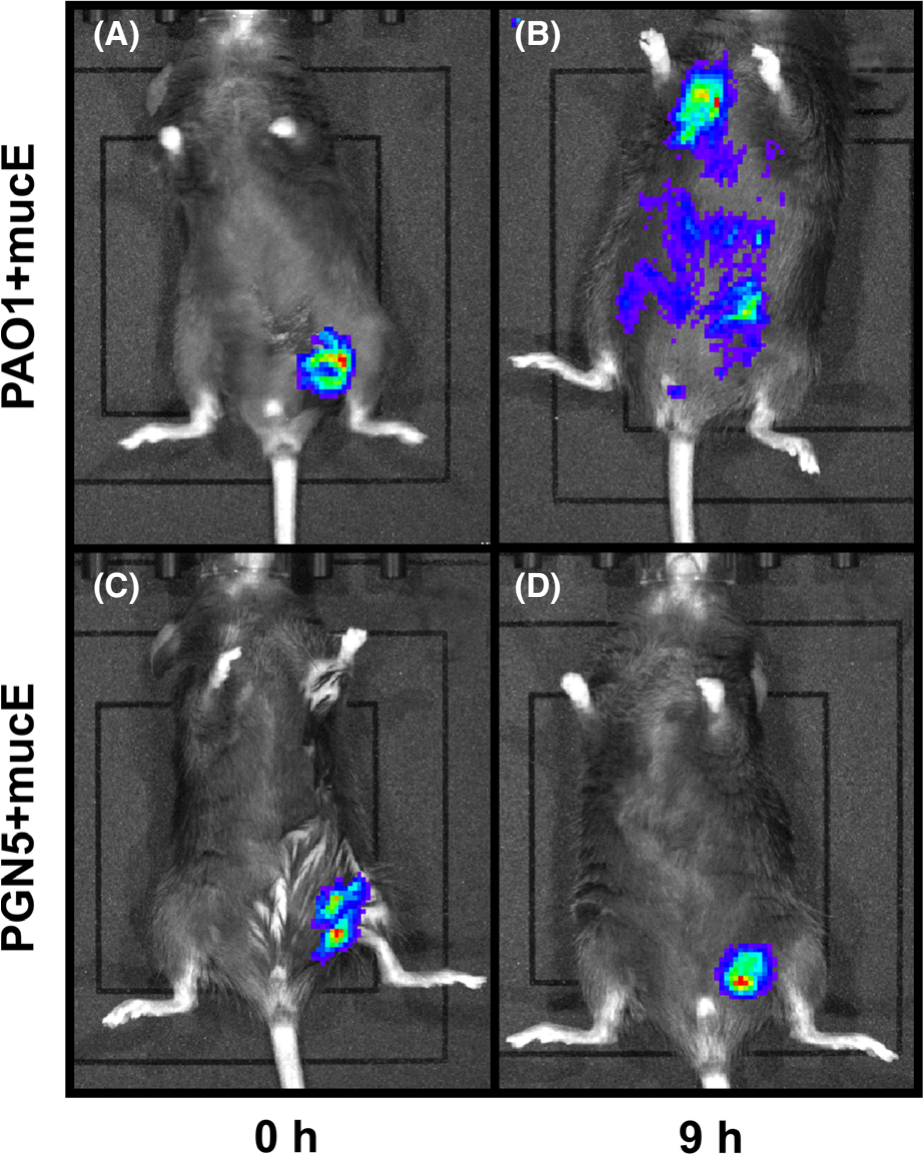
Images of mice injected with bioluminescent‐labelled *P. aeruginosa* strains. A,B. Mice injected with bioluminescent PAO1+mucE at A. 0 h post‐injection and B. 9 h post‐injection (*n* = 5). C,D. Mice injected with bioluminescent PGN5+mucE at C. 0 h post‐injection and D. 9 h post‐injection (*n* = 5). Note: different mice were imaged at each time point to avoid overdose of anaesthetic.

Since no mortality was observed of mice injected with PGN5+mucE, we wanted to determine whether cells of this strain might localize differently than VE2 cells within the mice post‐injection. To test this, we used the *luxCDABEG* operon to tag each strain with bioluminescence (Choi and Schweizer, 2006). VE2 and PGN5+mucE both carry gentamicin resistance genes, while the plasmids we used for labelling with bioluminescence required gentamicin sensitivity. Thus, we first incorporated the *luxCDABEG* operon into the chromosome of PAO1 and PGN5, and then introduced the pUCP20‐pGm‐*mucE* plasmid into each strain to induce alginate production and mucoidy, generating the bioluminescent strains PAO1+mucE (functionally equivalent to VE2) and PGN5+mucE. Intraperitoneal injection of C57BL/6 mice with bioluminescent PAO1+mucE showed either localization at the injection site or dissemination through the body, and lethality resulted in all mice injected (Fig. 5A and B). Conversely, localization at the injection site but no dissemination was observed with bioluminescent PGN5+mucE, and no mortality was observed in injected mice (Fig. 5C and D).

## Discussion

We have previously reported that overexpression of a novel activator of alginate synthesis, MucE, induces high and stable production of alginate in *P. aeruginosa* (Qiu *et al*., 2007). In this study, we report the generation of *P. aeruginosa* strain PGN5, which was engineered by sequentially deleting five pathogenic genes. These gene deletions were verified by biochemical or immunological assays to detect the absence or significant reduction in either the gene products or their activity, as well as through multiple DNA sequencing methods, including whole‐genome re‐sequencing. Analysis of the PGN5 genome not only verified deletion of the targeted five pathogenic genes, but also uncovered small insertions/deletions, SNPs and additional gaps in the genomic sequence compared to the PAO1 reference strain. The specific effects these changes to the genome have on the PGN5 strain are uncertain, but it is clear from our data that they do not affect the ability of PGN5 to make alginate. To test whether the five gene deletions attenuated the virulence of *P. aeruginosa*, we performed mouse infection model studies and challenged the animals with intraperitoneal injection of mucoid PGN5 (PGN5+mucE), its wild‐type counterpart VE2 (PAO1+mucE) or the FDA‐approved strain *E. coli* BL21 as a negative control. These strains were validated by PCR, phenotype and viable plate counts throughout the culture preparation and mouse injection procedure to verify strain authenticity and viable cell number. Inoculation of mice with PGN5 resulted in 0% mortality. In contrast, inoculation with VE2 resulted in 95% mortality while inoculation with BL21 resulted in 20% mortality. These results suggest that PGN5 is a highly attenuated *P. aeruginosa* strain. Indeed, our results suggest that PGN5 is less pathogenic and less toxic than the BL21 *E. coli* strain, which is FDA‐approved for production of biopharmaceuticals.

We also examined the dissemination of the bacteria after intraperitoneal injections in the mouse challenge experiments by using bioluminescence‐labelled mucoid PAO1 and PGN5 strains. All mice injected with bioluminescent PAO1 succumbed to the infection, while only about 50% exhibited systemic bacterial dissemination. In the other 50%, dissemination of bacterial toxins from the injection site most likely led to septic shock. In contrast, bioluminescent PGN5 was only detected at the injection site, no dissemination was apparent, and zero mortality was observed in these mice. This suggests that the invasiveness and toxin production associated with *P. aeruginosa* is either absent or reduced to a level that does not affect vitality in the PGN5 strain.

Importantly, overexpression of MucE in PGN5 induced stable *in vitro* production of alginate that was comparable to its wild‐type counterpart in chemical structure, physical characteristics of the alginate gel and total amounts produced. Thus, the genetic modifications in PGN5 have no measurable effect upon the alginate biosynthetic pathway. The viscosities of sodium alginate gels prepared from VE2 and PGN5+mucE alginate were nearly identical, as was the yield stress, suggesting that the gels have similar a tensile strength. HPLC chromatograms from alginate produced by VE2 and PGN5 were identical and rich in M residues. This is consistent with previous reports of bacterial alginates having relatively low G content (Tatnell *et al*., 1996; Moradali *et al*., 2015). While seaweed alginate composition varies between species and plant parts, it usually ranges from about 30–75% G content (Draget *et al*., 2005; Fertah *et al*., 2017). In general, alginate with higher G content boasts a higher tensile strength, and thus, it is a more desirable medium for some medical and industrial applications, such as 3D printing.

All commercially used alginate has been produced by extraction from brown seaweed. The process of harvesting and extracting alginate from seaweed is labour‐intensive, requiring harsh acid/base treatments and yielding large amounts of contaminated wastewater. In contrast, bacterial alginate can be extracted from the culture medium via ethanol precipitation, a procedure that does not require treatment with harsh chemicals. Because five pathogenicity gene sequences were completely deleted from different parts of the PGN5 chromosome, it is unlikely for this strain to revert to its original level of virulence. In contrast, other attenuated bacterial strains are often selected naturally as a result of point mutations; thus, the pathogenicity of these strains can be restored through reversion to wild type. PGN5 is also auxotrophic for aromatic amino acids and is unlikely to survive in case of accidental release into the environment. Thus, our data suggest that preparing alginate from cultures of PGN5 is a safe and highly reproducible method that is also an environmentally conservative alternative to using seaweed for the commercial production of alginate. Similar genetic manipulations of *Azotobacter* or other *Pseudomonas* species have the potential to generate other safe strains that stably produce alginate for use as platforms for microbial alginate production.

*Pseudomonas aeruginosa* is a well‐studied microbe, and many tools are available for the genetic manipulation of this organism. This makes PGN5 an attractive option for producing alginates with specific properties for distinct applications and possibly other recombinant proteins that are difficult to make in *E. coli*. Bioengineering this bacterium can be used to produce alginates with different physical properties, improving upon the characteristics of seaweed alginates and expanding applications for these specialty biopolymers beyond the current uses of alginate.

## Experimental procedures

### Bacterial strains, culture, plasmids and oligonucleotides

Bacterial strains and plasmids used in this study are listed in Tables 1 and 2. *P. aeruginosa* strains were either grown on *Pseudomonas* isolation agar (PIA) plates or in *Pseudomonas* isolation broth (PIB) at 37°C (Difco, Sparks, MD, USA). *E. coli* strains were cultured in Luria Broth (LB) or on LB with 1% agar at 37°C (Difco). When necessary, culture medium was supplemented with antibiotic as follows: gentamicin or carbenicillin at a concentration of 300 μg ml^−1^ for *P. aeruginos*a and 30 μg ml^−1^ for *E. coli*; kanamycin at a concentration of 50 μg ml^−1^ for *E. coli*. PGN5 was cultured with media supplemented with 1 mg/mL of the aromatic amino acids (Y, W, F) unless otherwise stated. Oligonucleotides used in this study are available upon request.

**Table 1:** Strains used in this study

Strain	Genotype	Relevant characteristics	Source
*Pseudomonas aeruginosa* strains			
PAO1	Wild‐type serogroup O5	Non‐mucoid, blue–green growth on PIA	Kropinski *et al*. (1979)
VE2	PAO1 with chromosomal fusion of P_Gm_‐*aacC1*‐*mucE*	Mucoid, blue–green growth on PIA	Qiu *et al*. (2007)
PA‐103	Serogroup O11	Positive for Exotoxin A secretion	Liu (1966)
PGN4	PAO1Δ*toxA*Δ*plcH* Δ*phzM*Δ*wapR*	Non‐mucoid, greenish colonies on PIA	This study
PGN5	PAO1Δ*toxA*Δ*plcH* Δ*phzM*Δ*wapR*Δ*aroA*	Non‐mucoid, white/tan colonies on PIA	This study
PGN5+mucE	PAO1Δ*toxA*Δ*plcH* Δ*phzM*Δ*wapR*Δ*aroA* pUCP20‐pGm‐*mucE*	Mucoid, white/tan colonies on PIA	This study
PAO1_*wbpL*_	PAO1Δ*wbpL*	Produces no O‐antigen	Rocchetta *et al*. (1998)
*Escherichia coli* strains			
BL21 (DE3)	F – *ompT hsdSB* (*rB‐ mB‐*) *gal dcm* (DE3)	FDA‐approved for production of biopharmaceuticals; deficient in ion and ompT proteases	Lucigen, Middleton, WI, USA
DH5α	F^−^ φ80dlacZΔM15 Δ(lacZYA‐argF)U169 deoR recA1 endA1 hsdR17(r_K_^−^ m_K_^+^) phoA supE44 λ^−^ thi‐1 gyrA96 relA1		Laboratory strain
TOP10	F‐ *mcrA* Δ(*mrr‐hsd* RMS‐*mcrBC*) φ80*lacZ*ΔM15 Δ*lacΧ*74 *rec*A1 *ara*D139 Δ(*ara‐leu*) 7697*gal*U *ga*lK *rps*L (Str^R^) *end*A1 *nup*G λ‐	Ideal for cloning and plasmid propagation	Invitrogen, CA, USA
SM10 (λpir)	thi recA thr leu tonA lacY supE RP4‐2‐ Tc::Mu1::pir Km^r^		Laboratory strain

**Table 2:** Plasmids used in this study

Plasmid	Relevant characteristics	Source
pEX100T‐NotI	*Pseudomonas* suicide vector with NotI restriction site fused into SmaI of pEX100T sacB oriT Cb^R^	Damron *et al*. (2009)
pRK2013	Helper plasmid for conjugation, Km^R^	Figurski and Helinski (1979)
pUCP20‐pGm‐*mucE*	*mucE* overexpression under gentamicin promoter, Gm^R^	Qiu *et al*. (2007)
pTNS2	Helper plasmid; does not replicate in *P. aeruginosa*. Cb^R^	Choi and Schweizer (2006)
pUC18‐mini‐Tn7T‐Gm‐*lux*	*Pseudomonas* suicide vector that carries *luxCDABE* operon for insertion into *att*Tn7 sites, Gm^R^	Choi and Schweizer (2006)
pFLP2	Facilitates recombination between FRT sites, Cb^R^	Choi and Schweizer (2006)

### Gene deletions

Retrieval and analysis of *P. aeruginosa* gene sequences were performed at the *Pseudomonas* Genome Database website: http://www.pseudomonas.com (Winsor *et al*., 2016). Five genes, *toxA, plcH*,* phzM*,* wapR* and *aroA,* were sequentially deleted from the chromosome of the wild‐type *P. aeruginosa* strain PAO1. The pEX100T‐NotI plasmid was used to mediate the in‐frame marker‐less deletion of each gene (Schweizer and Hoang, 1995; Damron *et al*., 2009). This plasmid carries the genes *ampR*, which confers resistance to carbenicillin, and *sacB* (*B. subtilis*), which provides sucrose sensitivity. Plasmid inserts used to delete *toxA*,* plcH*,* phzM* and *wapR* were generated by PCR amplification of 500–1000 bp of sequence directly upstream and 500‐1000 bp of sequence directly downstream of each target gene, followed by fusion of these DNA fragments via cross‐over PCR. The resultant PCR product was digested and ligated into pEX100T‐NotI. For in‐frame deletion of *aroA*, about 800 bp of upstream sequence adjacent to about 900 bp of downstream sequence of the target gene was synthesized, digested and ligated into pEX100T‐NotI by the company GenScript (Piscataway, NJ, USA). Each of the final plasmids was transformed into OneShot^TM^ TOP10 Electrocomp *E. coli* (Invitrogen, Carlsbad, CA, USA).

For each deletion, the pEX100T‐NotI plasmid carrying its specific insert was introduced into *P. aeruginosa* via triparental conjugation with the helper plasmid pRK2013. The target gene was deleted with a two‐step allelic exchange procedure. Briefly, homologous recombination between one site on the plasmid and its target site on the chromosome integrated the plasmid into the *P. aeruginosa* chromosome (i.e. a single‐cross‐over event). Single cross‐overs were selected by plasmid‐conferred resistance to carbenicillin and sensitivity to 10% (w/v) sucrose supplemented in PIA. Single cross‐overs were grown overnight in LB broth to allow for homologous recombination between the second site on the plasmid and its target site on the chromosome (i.e. a double‐cross‐over event), which removes the entire plasmid sequence along with the target gene sequence. Double cross‐overs were selected by sensitivity to carbenicillin and resistance to sucrose, indicating that the plasmid sequence had been removed. Sucrose‐resistant, carbenicillin‐sensitive colonies were sequenced to verify the in‐frame gene deletion.

### Gene sequencing

Sanger sequencing to confirm in‐frame deletions was performed by the West Virginia University (WVU) Genomics Core Facility (Morgantown, WV, USA). Whole‐genome re‐sequencing on these strains was performed by CD Genomics (Shirley, NY, USA). Genomic assembly of the data utilized the SPAdes microbial isolate assembler (Bankevich *et al*., 2012) followed by transfer annotation using PROKKA (Seemann, 2014). Homology‐based taxonomy assignment utilized PanGIA (Li *et al*., 2018), along with in‐house derived confidence level settings based on positive control DNA mixtures. Reference‐based analysis employed bowtie2 (Langmead and Salzberg, 2012) compared to the parental strain genome (GenBank accession number AE004091.2) with variant calling. bowtie2 settings include a 1000‐nt window and a 200‐nt step size. Whole‐genome sequences are available for PGN4 (GenBank accession number CP032540) and PGN5 (GenBank accession number CP032541). Pairwise genome comparison utilized the Artemis Comparison Tool for visualization (Carver *et al*., 2005).

### Western blot for Exotoxin A

A Western blot was used to verify the deletion of *toxA* in PGN5 by the absence of the Exotoxin A product. Cells from 24‐h broth cultures of *P. aeruginosa* strains PAO1, Exotoxin A‐positive PA103 and PGN5 were removed by centrifugation. Supernatant was filter‐sterilized with a 0.2 μm syringe filter and concentrated with an Amicon Ultra‐15 Centrifugal Filter Unit with a 10‐kDa cut‐off (Sigma, St. Louis, MO, USA) according to the manufacturer's instructions. Total protein concentration of the supernatant (extracellular fraction) was quantified with the bicinchoninic acid assay (BCA) using a bovine serum albumin (BSA) standard (Pierce, Rockford, IL, USA). For Coomassie staining, equal amounts of protein (60 mg) were run with SDS‐PAGE on a TGX 4‐20% gel (Bio‐Rad, Hercules, CA, USA), stained with Coomassie R‐250 (Protea Biosciences, Morgantown, WV, USA) and imaged on a ProteinSimple Fluorochem M (San Jose, CA, USA). For Exotoxin A western blot, samples with equal total protein concentrations (0.05 μg/μL) were run on the ProteinSimple Wes automated Western blot system using the 12–230 kDa separation module and the 8 × 25 capillary cartridge (San Jose, CA, USA). Exotoxin A was detected with a polyclonal rabbit anti‐Exotoxin A antibody (Sigma, St. Louis, MO, USA) diluted 1:200 and the ProteinSimple anti‐rabbit detection module (San Jose, CA, USA).

### Analysis of haemolytic activity

To verify deletion of *plcH*, haemolytic activity of *E. coli* strain BL21 and *P. aeruginosa* strains PAO1, VE2 and PGN5 was assessed by culturing bacterial strains for 48 h on blood agar (trypticase soy agar with 5% sheep blood; Remel, Lenexa, KS, USA). Before inoculation, plates were treated with a 1 mg ml^−1^ solution of aromatic amino acids and dried. Plates were imaged for the presence or absence of clear plaques indicating haemolysis surrounding bacterial growth.

### Extraction and quantification of pyocyanin

To verify deletion of *phzM* in PGN5, a two‐step extraction procedure with chloroform and HCl was used to recover and quantify the secreted pyocyanin pigment (Essar *et al*., 1990). Briefly, cells from 24‐h broth cultures of *P. aeruginosa* strains PAO1 and PGN5 were removed by centrifugation and the supernatant was filter‐sterilized with a 0.2‐μm syringe filter. Pyocyanin was extracted from 7.5 ml samples with 4.5 ml of chloroform. Pyocyanin was re‐extracted from 3 ml of the chloroform layer with 1.5 ml of 0.2 M HCl to give a pink colour. The absorption at 520 nm (A_520_) of the aqueous HCl layer was measured with a SmartSpec™ 3000 spectrophotometer (Bio‐Rad, Hercules, CA, USA), and pyocyanin concentration was calculated as follows:

$$A_{520}\times 17.072\times 1.5=\text{pyocyanin concentration}\;\left(\text{in}\;\mu l\;{\text{ml}}^{-1}\right).$$

### LPS extraction, silver staining and Western blot for O‐antigen

To verify deletion of *wapR*, lipopolysaccharides (LPS) were analysed for the presence of O‐antigen. LPS was extracted using the Hitchcock and Brown method (Hitchcock and Brown, 1983), resolved by electrophoresis on 12% SDS‐PAGE and stained for visualization using an ultrafast silver staining method (Fomsgaard *et al*., 1990). For Western immunoblotting, LPS was transferred onto BioTraceNT nitrocellulose membranes (Pall) and detected with a 1:1 mixture of monoclonal antibodies MF15‐4 (OSA specific) and 5c‐7‐4 (inner core specific) overnight at room temperature, followed by incubation with an alkaline phosphatase‐conjugated goat anti‐mouse Fab_2_ secondary antibody (Jackson ImmunoResearch, West Grove, PA, USA). The blots were developed using nitroblue tetrazolium (NBT) and 5‐bromo‐4‐chloro‐3‐indolylphosphate (BCIP) as described previously (Blake *et al*., 1984; de Kievit *et al*., 1995).

### ELISA for AroA

An enzyme‐linked immunosorbent assay (ELISA) was used to verify deletion of *aroA* by determining the absence of the 3‐phosphoshikimate 1‐carboxyvinyltransferase product. A 96‐well untreated plate was coated with equal amounts of carbonate buffer and of broth culture of the *P. aeruginosa* strains PAO1 and PGN5. The plate was then incubated for 2 h at 37°C and blocked in skim milk overnight. A polyclonal rabbit anti‐AroA primary antibody (LifeSpan BioSciences, Inc., Seattle, WA, USA) and a polyclonal goat anti‐rabbit IgG secondary antibody (Sigma, St. Louis, MO, USA) were used to detect 3‐phosphoshikimate 1‐carboxyvinyltransferase. Samples were incubated in 1‐Step™ Ultra TMB ELISA Substrate Solution (Thermo Scientific, Rockford, IL, USA), followed by addition of acid stop solution (R&D Systems, Minneapolis, MN, USA). The absorbance was immediately measured at 450 nm (A_450_) on a Spectramax^®^ i3x microplate reader (Molecular Devices, Sunnyvale, CA, USA). Absorbance reported was the relative absorbance detected between PAO1 and PGN5. Reported values represent the mean ± SD of three independent experiments. Two‐tailed Student's t‐test was used to determine statistical significance.

### Growth assays

To quantify rescue of growth of the PGN5 strain by aromatic amino acid supplementation, PIB supplemented with gentamicin was inoculated with a single colony of the *P. aeruginosa* strains PGN5+mucE or VE2 and grown at 37°C until reaching an A_600_ of 1, as measured on a SmartSpec™ 3000 spectrophotometer. The seed cultures were then used to inoculate PIB supplemented with either 0, 0.5 or 1 mg ml^−1^ of aromatic amino acids (Y, F, W). Cultures were grown for 72 h at 37°C, and A_600_ measurements were taken every 24 h to estimate the total number of cells in each culture. The results presented represent three independent experiments.

### Carbazole assay

The carbazole assay is a standard uronic acid detection method that was used to quantify the amount of alginate produced in culture. The *P. aeruginosa* strains VE2 and PGN5+mucE were grown on PIA for 48 h at 37°C. Alginate and cells were collected with 0.85% NaCl. Cell suspensions were incubated in sulfuric acid/borate solution and carbazole at 55°C. As carbazole reacts with uronic acid, the solution turns a pink to purple colour. Absorbance at 530 nm (A_530_) was measured on a SmartSpec™ 3000 spectrophotometer. The concentration of alginate (μg/ml OD^−1^_1600_) was calculated using a standard curve generated with purified mannuronic alginic acid (Sigma, St. Louis, MO, USA).

### Collection of alginate

*Pseudomonas aeruginosa* strains VE2 and PGN5+mucE were cultured for 72 h in 1 l of PIB or PIB supplemented with 1 mg ml^−1^ of aromatic amino acids. Alginic acid was precipitated with three volumes of ethanol, vacuum‐filtered through a Twill Dutch weave wire cloth (5 μm; Dorstener Wire Tech, Spring, TX, USA) and dried in a vacuum oven. The dried weight of alginate collected from VE2 and PGN5+mucE strains was presented as the mean ± SD of five independent experiments.

### Compositional analysis of alginate via HPLC

Determination of the M:G sugar ratios was performed by HPLC analysis using an adapted pre‐column derivatization of hydrolysed alginate with 1‐phenyl‐3‐methyl‐5‐pyrazolone (PMP) (Honda *et al*., 1989). Seven mg of dried alginic acid was suspended in 962 μl of water and 38 μl of trifluoroacetic acid (TFA) and hydrolysed in a sealed vial at 100°C. After 4 h, the pH of the samples was adjusted to 9.5 and 0.5M PMP in methanol (0.15 ml) was added. The samples were incubated at 70°C for 1.5 h with shaking at 1000 r.p.m. The pH was adjusted to 6.5, and excess PMP reagent was removed by repeated extraction with chloroform prior to analysis. The M/G ratios were determined by HPLC using an Agilent Infinity II HPLC system equipped with a 1260 quaternary pump (G7111B), vial sampler (G7129A), multicolumn thermostat (G7116A), diode array detector (G7115A) and Openlab CDS ChemStation Edition (v. C.01.08 [210]) software. Chromatographic separation was performed using an Agilent Eclipse Plus C18 column (4.5 × 150, 3.5 μM) and a mobile phase consisting of 0.1 M phosphate buffer (pH 6.7) with acetonitrile at a ratio of 82:19 (v/v, %). The column temperature was maintained at 25°C, and elution of the derivatized M/G monomer at 1 ml min^−1^ was detected at 245 nm. As a control, commercially available alginate from seaweed (FMC Protanal CR 8133, Wilmington, DE, USA) was prepared and run along with *P. aeruginosa* alginate samples.

### Physical property testing of alginate

To test the physical properties of bacterial alginates, sodium alginate gels were prepared from dried alginic acid. Briefly, alginic acid was dissolved in NaOH pH 8.5–9 to form sodium alginate, which was then precipitated with ethanol, filtered and dried in a vacuum oven. Sodium alginate gels (2% w/v) were prepared by dissolving sodium alginate in distilled water. Sodium alginate gels were prepared for comparison from seaweed alginate samples purchased from Sigma: alginic acid sodium salt, from brown algae (Sigma 2158; catalogue number A2158) and alginic acid sodium salt (Sigma 180947; catalogue number 180947). Viscosity and yield stress were measured on a HAAKE MARS I Rheometer (Thermo Scientific, Karlsruhe, Germany) using preset parameters. Results are presented as the mean ± SD of 3–5 independent experiments. Analysis and calculations were performed with RheoWin Software (version 4.82, Thermo Scientific).

### Bacterial pathogenicity in mice

*Pseudomonas aeruginosa* strains PGN5 and VE2, and *E. coli* strain BL21 were grown in LB broth to generate frozen stocks for injection into C57BL/6 mice. Cell cultures were concentrated to 2.5 × 10^9^ cells per ml, flash‐frozen in liquid nitrogen and stored at −80°C. The cultures used were strain‐verified by PCR and phenotype, and cell concentrations were verified by viable plate counts before freezing and before mouse injection. A total of eighty 10‐ to 12‐week‐old mice were divided into four groups of 10 males and 10 females. Each group received 200 μl intraperitoneal injections of one of the following: PBS, 5 × 10^8^ cells of the *P. aeruginosa* strains VE2 or PGN5+mucE, or 5 × 10^8^ cells of the *E. coli* strain BL21. After injection, mice were monitored for mortality every 3 h for 72 h and then every 12 h for 7 days.

### Localization of bioluminescent bacteria in mice

PAO1 and PGN5 were marked with bioluminescence using the method and plasmids developed in the Schweizer laboratory (Choi and Schweizer, 2006). The pUC18 mini‐Tn7T‐Gm‐*lux* plasmid was used, which allows for insertion of the *luxCDABE* operon, along with an FRT‐flanked gentamicin resistance cassette into a neutral site downstream of the *glmS* gene. Briefly, electrocompetent PAO1 and PGN5 cells were prepared with 300 mM sucrose and electroporated with the pUC18 mini‐Tn7T‐Gm‐*lux* and pTNS2 plasmids. Pure stocks were generated from resultant gentamicin‐resistant and bioluminescent colonies. The pFLP2 plasmid was used to remove the gentamicin resistance cassette. Final stocks used for mouse injection were PCR‐verified, bioluminescent, plasmid‐cured, and gentamicin and carbenicillin‐sensitive. For mouse injection, stocks were prepared and injected as described above. Mice were imaged on an IVIS Lumina XRMS (PerkinElmer, Waltham, MA, USA) every 6 h for 18 h and monitored for 4 weeks. By 18 h post‐injection, bioluminescence was only detected at the injection site of all mice.

### Statistical analyses

graphpad prism 7.02 for Windows (GraphPad Software, La Jolla, CA, USA) was used to generate graphs and perform statistical analyses.

## Conflict of interest

RMN and HDY are co‐founders of Progenesis Technologies, LLC, which develops, produces and markets bacterial alginates.
